# Children’s fear of needle injections: a qualitative study of training sessions for children with rheumatic diseases before home administration

**DOI:** 10.1186/s12969-020-0406-6

**Published:** 2020-02-07

**Authors:** Kari Sørensen, Helge Skirbekk, Gunnvald Kvarstein, Hilde Wøien

**Affiliations:** 1 Department of Nursing Science, University of Oslo, Oslo, Norway; 2 Department of Pain Management and Research, Oslo University Hospital, Oslo, Norway; 3 Department of Undergraduate Studies, Lovisenberg Diaconal University College, Oslo, Norway; 4 Department of Health Management and Health Economics, University of Oslo, Oslo, Norway; 5 Department of Clinical Medicine, the Arctic University of Norway, Tromsø, Norway; 6 Division of Emergencies and Critical Care, Oslo University Hospital, Oslo, Norway

**Keywords:** Fear of needle, Subcutaneous injection, Home administration, Rheumatic disease, Juvenile idiopathic arthritis, Qualitative research, Video observation

## Abstract

**Background:**

Treatment of rheumatic diseases in children often includes long-term needle injections, which represent a risk for refusing medication based on potential needle-fear. How nurses manage children’s fear and pain during the initial educational training session of subcutaneous injections, may affect the management of the subsequent injections in the home settings. The aim of this study was to explore how children expressed fear and pain during these training sessions, and how adults’ communication affected children’s expressed emotions.

**Methods:**

This qualitative explorative study used video observations and short interviews during training sessions in a rheumatic hospital ward. Participants were children between five and fifteen years (*n* = 8), their parents (*n* = 11) and nurses (*n* = 7) in nine training sessions in total. The analysis followed descriptions of thematic analysis and interaction analysis.

**Results:**

The children expressed fears indirectly as cues and nonverbal signs more often than direct statements. Three children stated explicit being afraid or wanting to stop. The children worried about needle-pain, but experienced the stinging pain after the injection more bothersome. The technical instructions were detailed and comprehensive and each nurse shaped the structure of the sessions. Both nurses and parents frequently offered coping strategies unclearly without sufficient time for children to understand. We identified three main adult communication approaches (*acknowledging*, *ambiguous* and *disregarding*) that influenced children’s expressed emotions during the training session.

**Conclusions:**

Children’s expression of fear was likely to be indirectly, and pain was mostly related to the injection rather than the needle stick. When adults used an acknowledging communication and offered sufficient coping strategies, children seemed to become involved in the procedure and acted with confidence. The initial educational training session may have a great impact on long-term repeated injections in a home setting by providing children with confidence at the onset.

## Background

Needle related fear is common, particularly in children [1]. It may impede vaccination and treatment programs based on medical injections [2, 3]. Children with rheumatic diseases like juvenile idiopathic arthritis (JIA) are especially vulnerable, as they are often treated with long-term subcutaneous injections of Disease-modifying antirheumatic drugs (DMARDs) and biologics [2, 4]. In one study, adults who had suffered from JIA for 30 years had lower physical function, lower health related quality of life and more pain than the general population [5]. Targeted medical treatment with DMARDs and biologics may improve the quality of life of JIA patients and may even bring the disease into remission [6]. However, the risk of relapse is significant and requires ongoing medication for years [7].

At home, subcutaneous injections are mainly administrated by parents or by children themselves. However, high levels of fear are associated with perceived pain during needle procedures [8], and the need for ongoing injections is a substantial stress factor for children and their families [4]. Therefore, alleviating fear is important [3]. Non-pharmacological strategies may improve children’s coping [9–14], while some types of adult communication, such as reassurance, are associated with increased distress [15–17]. Distress describes several negative experiences like fear, pain and anxiety [18]. Historically, children have been ignored as active participants in *doctor-parent-child* communication [19] and are still rarely included in shared decision making [20]. In general, there is a lack of attention on children’s emotions during medical consultations [21].

Clinical guidelines for the management of needle related fear and pain in children are mostly based on research into vaccination and venepuncture [22, 23]. Children with rheumatic diseases, who require repeated injections over time probably experience needle sticks differently from healthy children, who receive a limited number of vaccines. Thus, research on children in different contexts has been recommended to find methods to manage children’s pain and suffering [24]. The way nurses relate to children and parents during training sessions and how they manage fears and worries may affect how injections are subsequently managed in home settings. Studying these training sessions may provide valuable knowledge for future clinical and educational recommendations. Children’s participation in research is valuable, but it is essential to assess their vulnerability during the first medical injection carefully [25, 26].

The aim of this study was to explore children’s expressions of fear and pain during training sessions for the home administration of subcutaneous injections. We also aimed to explore how nurses’ and parents’ communication affected children’s expressed emotions.

## Methods

### Design

We chose a qualitative explorative design with an ethnographic approach, because it allowed us to describe and understand a phenomenon in a specific context [27]. We used video observation and subsequent short interviews with participants to obtain detailed data of ongoing communication and interactions between children, parent(s) and a nurse within a natural setting [28, 29].

### Setting and participants

The study took place at a Norwegian university hospital that offered treatment to children with rheumatic diseases. When children were diagnosed and home medication prescribed, nurses educated children and their parents on how to self-administer needle injections. Usually, the education and first injection took place during a session in the paediatric ward, while subsequent injections were performed at home.

Participants in this study were nurses, children and their parents. To be included, nurses had to engage in patient education as a regular task during their daily work. Children had to be between five and fifteen years and in need of education on subcutaneous injections of DMARDs and biologics. Children with prior experience of injections were included if they needed a new education session due to new medication. Participants within each session represented an interactive unit in the social process studied, hereafter termed *a case* [27].

### Data collection

Data was collected between June 2017 and December 2018. We used purposive sampling, which allowed us to choose participants that acted in the context in which we were interested [27]. The first author (KS) informed all nurses in the ward about the study prior to its onset. Nurses were invited to reflect upon positive and challenging consequences of participation during formal and informal meetings within the study period. A coordinating nurse assisted the researcher and ensured that only nurses willing to participate were connected with children (and parents) who met inclusion criteria. Participating nurses gave brief information about the study to children and parents identified as potential participants. If they agreed, then KS was contacted to provide more detailed information before children and parents consented to participate.

The observation procedure was pilot tested by KS during a training session without video recording. Video recording is considered an ideal method of gathering data in a natural setting [28] and causes minimal disturbance of the child-adult interaction. Two video cameras were placed in the room to capture a close-up of the child’s face and a wide screen shot to obtain a full view of the training scene [28]. The use of GoPro cameras made it possible to prepare camera arrangement quickly. Video recordings began at the onset of the procedure and were stopped when nurses signalled that they were finished. The observer (KS) was present during the whole session and took field notes to contextualise the interaction [27]. It was possible to turn the video cameras off if they caused an extra burden for the child. In one case, participants changed places, making it difficult to view the child’s face; however, KS could still observe the child’s facial expression. A short interview with participants was completed immediately after the procedure in which they reflected on the experience of being filmed, and children were asked about their anticipatory fear of needles.

### Data Analysis

The analysis drew on descriptions of thematic analysis (TA) [30, 31] and interaction analysis [32]. After following the six phases of TA, a systematic presentation of the findings with specific descriptions of the children’s expressions of fear was created. To conduct an in-depth exploration of the interaction between nurse, child and parent(s), we carefully searched for *events* during which children showed distressed behaviour and looked for patterns that influenced changes in their expressed fear and pain.

All verbal conversations in the video recordings were transcribed by the first author (KS). Nonverbal signs and behaviour were marked. Fields of particular interest were underpinned and main impressions documented. All videos were viewed and reviewed by all authors. Some parts of the videos were studied during group sessions. Then, KS and HW coded the data. We were particularly interested in how children expressed negative emotions like fear and pain and how nurses and parents responded. The process used to identify emotions expressed indirectly and nonverbally was inspired by prior research in this field that used the Verona Coding Definitions of Emotional Sequences (VR-CoDES), a system for identifying patients’ expressions of emotional distress during medical consultations [33, 34].

**Fig. 1:**
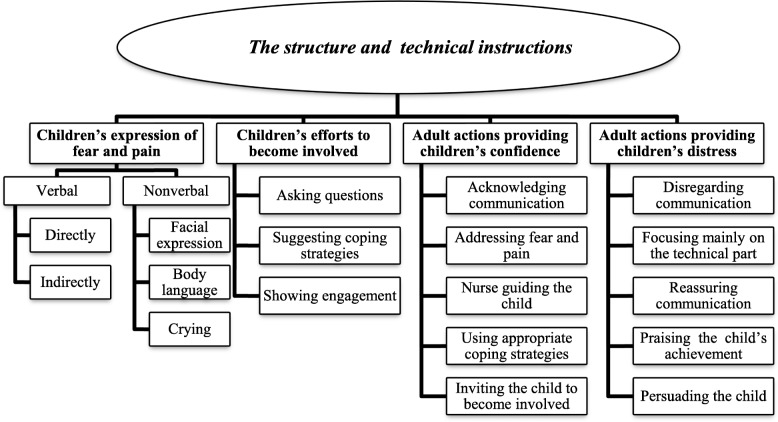
Thematic map. The thematic map shows the four main themes and 20 defined subthemes used to condense verbal and nonverbal communication and to describe coded actions and interactions between participants in the training sessions. The structure and technical instructions defined the context

Participants’ verbal and nonverbal communication was identified using a total of 67 codes. These were grouped into preliminary themes. All authors contributed to an ongoing reflexive clarification of themes to ensure that they worked well in relation to the data and research questions. In this phase of the analysis, we aimed to move from a summative position to an interpretative orientation and to develop a final thematic map (Fig. 1). We used the software tool NVivo 11 to obtain a systematic organisation and to perform the analysis [35]. NVivo’s functionalities of viewing *coding stripes*, *comparing nodes* and *exploring hierarchy charts* were useful when looking for patterns across the dataset.

### Trustworthiness

Generalisation in qualitative research is based on identifying social processes rather than from the representative sampling of individuals [27]. Credibility was achieved by describing participants’ conversations and behaviour, including quotations. Confirmability was ensured by involving co-authors in all steps of the analytic process and by presenting the analytic steps from raw data to the results. Transparency was sought through detailed descriptions of the research process, allowing the reader to assess the research practice. To validate the fact that the presence of the researcher did not interfere with the procedure, each nurse was asked if the session had taken place as normal [36]. By providing sufficient contextual information about the study, we aimed to ensure transferability [37]. Triangulation between data from different sources, like field notes from the session and the short interview, contributed to its validation [27]. Consolidated criteria for reporting qualitative research (COREQ) were used as a guide to report this study [38].

## Results

A total of eight children, seven nurses and eleven parents participated in nine cases. Characteristics of the cases have been described in Table 1.

**Table 1:** Characteristics of the cases

Videos	Case 1	Case 2	Case 3	Case 4	Case 5	Case 6	Case 7	Case 8	Case 9
Child	Girl^a^	Girl^b^	Boy	Girl^c^	Girl	Boy^d^	Boy	Girl	Girl
Age (years)	10	5	14	15	8	15	15	12	12
Parents	two	one	one	two	two	one	one	one	one
Nurse	A	B^e^	C	B	D	E^e^	F	E	G
Performing injection	Nurse	Parent	Boy	Girl	Nurse		Nurse	Parent	Nurse
Session length	31 min	13 min	27 min	6 min	10 min	13 min	7 min	24 min	20 min
Medication (syringe/pen)	Enbrel (syringe)	Enbrel (syringe)	Enbrel (syringe)	RoActemra (pen)	Metex (pen)		Humira (pen)	Benepali (syringe)	Benepali (syringe)

^a^Diabetes type I, treated with Insulin pump

^b^Metex s.c a few times

^c^Injections by parents year ago, intravenous infusions before new education of s.c. injections

^d^The same boy in case 6 and 7

^e^Nurse B and E participated in two cases

All nurses were female with a mean age of 28.9 (26–34) years. Of the total sample, six had worked as registered nurses at this ward for less than one year and two nurses implemented a training session for the very first time. Four nurses had prior education into music, psychology, pedagogy or law. Those who refused to participate included one child and two of 20 available nurses. Findings suggested four main themes of interest, which have been summarised in a thematic map (Fig. 1)

.

A total of 20 defined subthemes were used to condense verbal and nonverbal communication and to describe coded actions and interactions. The structure of the session and the technical instructions given by nurses defined the context of these actions and interactions.

### Structure and technical instructions

All sessions were carried out in patients’ bedrooms, to which nurses brought the necessary equipment. Occasionally, the only table in the room was overloaded with the child’s and parents’ personal belongings, so medical equipment was placed in between these. Specific equipment used to distract children or help them cope during painful procedures was not available in the room. However, some children had their own toys or mobile phones available.

Nurses were responsible for safety during this complex procedure, that included medical, technical and hygienic aspects and to perform the session in a shortage of time. The technical information they provided was comprehensive and detailed (Table 2). Written or drawn age appropriate information was unavailable, so nurses sometimes offered to write down instructions or recommended that children and their parents watch videos on YouTube. In this study, two children had watched an educational video in advance of the session and were disappointed when they realised that the syringe differed from the pen for which they had prepared. Most children were invited to rehearse the self-administering of injections with the equipment and to poke needles into rubber skins, but the practice kit was sometimes different from the one they would use at home. Preparing for prefilled pens was easier and safer, but containing too large doses for children, only three children were offered this device. Thus, parents (and children) had to learn how to transfer a small dose from one syringe to another.

**Table 2:** Detailed and comprehensive technical information

Codes	Illustrating quotations
Equipment and technique	*“You will hear a “click” when you push the bottom and then the chamber will be yellow”*
	*“Your child is going to have 0,35 ml and this contain 1 ml”*
	*“You must put it in an angle of 45 or 90 degrees”*
	*“You must squeeze up the skin and inject”*
	*(All cases)*
Warning	*“Watch out for sharp needles”*
	*(Case 1,3)*
Hygiene	*“You must wash the skin with this swab, to make sure it’s clean”*
	*(All cases)*
Drug information:	
• Storage	*“You must take the medicine out of the refrigerator, 15 min before you inject it”*
• Waste	*“You will get a yellow box from the pharmacy”*
	*(Case 3, 4, 6, 8)*
Use of aids	*Syringes, pens, rubber skin (Case 1, 3, 5, 6, 8, 9)*

In four of the nine cases, the injection was given by the nurse, leaving the children and parents without the experience of administering an injection. No additional routine appointments for training sessions were scheduled.

Shortly after the training session we asked the nurses about their experiences of being filmed. The nurses stated being a bit nervous attending a video observation, but claimed they quickly got used to the camera and acted as normal as for other daily procedures at the ward.

### Children’s expression of emotions

In this study, seven of the eight children showed obvious signs of fear or worry about the needle injection. Their expression of fears varied from slight excitement to severe anxiety. Fear was mostly expressed indirectly or nonverbally. Only three children stated explicitly that they were afraid or refused to continue the procedure. Verbal expressions of fear have been exemplified in Table 3.

**Table 3:** Children’s verbal expression of fear

Codes	Illustrating quotes and behaviour
Directly expressing fear	*“I am still afraid”*
Denying	*“I don’t want to”*
Asking for time	*“I need to practice”, “wait”*
Being quiet, spend time	*(Saying nothing or speak with low voice for a long time)*
Trying to understand	*“And it’s not possible to take it slowly either”*
Challenging the adult	*“You didn’t make to get it ready in time” (counting fast to 20)*
Bodily symptoms	*“I may be sick when taking blood samples – that happened once”*
Using onomatopoeia	*“Oh”, “Ahaa”, “Wow”*
Repeating neutral words	*“I must burst, burst, burst, burst …*” *(said with a raising tone in the voice)*

Children showed nonverbal signs, including a slight smile, insecure laughter, scratching themselves, intense wriggling, sitting stiffly in the chair, keeping their hands in front of their face, leaning on their parents, holding their hands on their stomach or shivering, moaning or crying with different strengths. Adolescents typically communicated fear nonverbally and consented to the injection despite being afraid, as exemplified in the following conversation:

*Nurse: “As long as you find a technique that is okay for you … .”*

*Child gasps, wriggles in the chair*

*Nurse: “Shall we fill a real syringe then?”*

*The child nods (Case 3)*

This child’s anxiety was verbalised in the short interview after the procedure as follows:

*Researcher: “How much did you worry on a scale from 0 to 10, where 0 is no worry and 10 is the most worrying thing you might think of?”*

*Child: “Seven, I didn’t know what to expect” (speaking with clear voice)*

*Nurse: “But you looked very calm, even being so worried?” (Nurse looking surprised)*

*Child: “Yes, it’s inside of me” (Case 3)*

Children’s fear did not depend on whether the injection was given by syringe or pen, as the device was new for each child. The three children with prior experience with injections showed different levels of fear; one stated being a bit worried (3–4 on a scale from 0; no worries at all to 10; worst worry imagined), one stated several times being afraid and denied to have the injection (screamed load), and one claimed not being worried at all, looking forward to skip the current intravenous infusions at the hospital every fourth week. In total, three children cried before the injection. Of these, two explicitly and repeatedly said that they were afraid and did not want to take part in the procedure. These children sat unmoving during the injection, but their facial expressions looked sad, and they searched for physical support from their parents. Nevertheless, the only child denying fears showed a great relief after the injection and shouted a loud “*yeah*” *(Case 4)*. Most children reported that the feared needle puncture was less painful than the subsequent stinging pain. Nevertheless, they would have liked to be prepared for this pain. It was one child that screamed out and cried for several minutes.

All children tried to become involved during the sessions, usually by being occupied with a task. They behaved in a more relaxed manner when playing with the equipment and their engagement sometimes increased when they received less attention from adults. For example, one child was lying in bed showing little interest but practiced more intensely when the nurse gave her attention to the parent (*Case 6*). Another child had two breaks during the session, because the nurse needed additional equipment, and the child utilised the breaks to share worries with the parent. When the nurse returned, the child asked directly if the needle would hurt.

The nurse replied:

*“Some think it’s painful and some don’t. What people often feel unpleasant, if it hurts, it’s not the needle stick itself but a slight stinging pain afterwards” (Case 9)*

Even though this child was afraid, she looked confident after the injection, stating that it was less painful than feared.

Children often asked practical questions about the injection site and whether to be aware of bubbles, or they tried to figure out what it would feel like. Three children were prepared with topical anaesthetic and examined their thigh to find a place where it would not hurt. The tone and volume of their voices rose as the hope of a pain-free injection increased and lowered when they felt something touching their skin.

### Adults’ responses to children’s fear and pain

Conversations during sessions usually included instructions from the nurse to the child and parent and practical questions from parents. Frequently, nurses did not ask children and parents about what they needed to learn or mapping questions related to prior experiences of fear and pain. Communication about fear and pain was sometimes initiated by nurses but was more often a response to children’s nonverbal or verbal expressed emotions. Nurses suggested choices on positioning, holding hands and watching, but they generally missed to explain why these suggestions might help children cope during the procedure. All nurses praised the child (and parents) for their skills and willingness to perform the injection. The technical part of the procedure required much attention, and children’s attempts to become involved and understand what was going on were not always perceived. We identified three main adult communication approaches (*acknowledging*, *ambiguous* and *disregarding*) that influenced children’s coping (Table 4).

**Table 4:** Examples of three different main approaches by the adults towards the child’s fear

1. Acknowledging communication		
Nurse activities	*Communication and interaction*	Child response
Addressing fear	*Nurse: “So, what you might do when I give you the shot; is to choose to look at it, or you can look at mummy or daddy, but it might hurt, right?”*	Becoming engaged
Suggesting coping-strategy; time to reflect	*Child: “Yes” (nods)*	
	*Nurse: “When you feel the needle stick, you might squeeze your mother’s hand as hard as you feel it”*	
Guiding the child (and parents)	*Child: “And I can think that when its finished; it’s finished, and it’s a week until next time..”*	
	*Nurse: “Yes”*	Showing confidence
	*Child:* “*… and then, it might not hurt so much …*” (*Case1*)	
2. Ambiguous communication		
Nurse activities	*Communication and interaction*	Child response
Addressing fear	*Child: “Shows an insecure smile (non-verbal fear)*	
	*Nurse: “I do understand if you worry about the needle-injection, it might hurt”*	Not time to become engaged
Suggesting coping-strategy; unclear, no time to reflect	*Nurse: “Do you want to look at it or to mummy or …*.?*”*	
	*Child: “I don’t know”*	
	*Nurse: “You do as you like, what you think is best – okay? (no answer) here it is; just like a pen don’t you think? The medication is in here; not so much - and you can see that this is the one getting yellow – right? (hearing the nurse take a deep breath) -, then it’s nice and quiet”*	
Taking control	*Child: Whispers something impossible to hear*	
	*Nurse: “Shall we just have it done? Yes, I will give it here”*	Crying
	*Child: “Oh – (cries quietly)”*	Surrendering
Praising the child	*Nurse: “Do you want me to count before I do it?*	
	*Child: (no answer)*	
	*Nurse:* (Gives the shot). *There we are (with laud voice) – very brave!”*	
Talking about the experience	*Child: “Yes”*	Showing relief and embarrassment (confused)
	*Nurse: “How does it feel? Was it painful?”*	
	*Child: “It didn’t hurt so much”* (*Case 7*)	
3. Disregarding communication		
Nurse activities	*Communication and interaction*	Child response
Reassuring	*Child: “The needle stick will hurt”*	Continuing to express fear
	*Parent 1: “It will be over soon”*	
	*Child: Speaks in a very low voice*	
Suggesting coping strategy; unclear, and persuading	*Nurse: “You will hardly notice anything”*	
	*Child: “Yes, but I don’t dare”*	
	*Parent 2: “Come on, you can hold on to me”*	Crying
	*Child: “I don’t dare” –(cries)*	Protesting
	*Parent 2: “Breath”*	
	*Child: “I don’t want to” – (cries)*	
Offering a prize	*Nurse: “I will find you a prize afterwards”*	
	*Child: “I don’t want to” – (cries softer) (Case5)*	Surrendering

#### Acknowledging communication

In some cases, nurses acknowledged children’s emotions and offered enough time to reflect on them (Table 4). These nurses managed to translate indirectly stated worries to an explicit fear and suggested possible coping strategies. Children acted more relaxed with increased engagement. In these cases, nurses and children reached a mutual understanding on the enactment of the procedure, and children expressed confidence and less pain than expected. In the cases in which nurses used acknowledging communication, parents were supportive of the communication between nurse and child. In one case, the parent mediated the communication, particularly when it came to a break (Case 9).

#### Ambiguous communication

In some cases, adults were aware of their children’s fear but did not address it sufficiently. Coping strategies were suggested, but this was done too late or after the child had become distressed. Nurses in these cases made efforts to guide children through the procedure, but they failed to reach a mutual understanding (Table 4). During these procedures, both nurses and parents mainly used reassuring communication, that is, “*You won’t feel much pain*”, “*The needle is thin*” or “*This will do you good*”. One child intended to inject the medication herself but stated explicitly that she was afraid and denied to watch. The nurse continued to reassure her, even when her distress increased. In addition, parents’ activity increased, as they offered a mix of comfort, reassurance, physical support and slight attempts at distraction. Afterwards, nurses praised these children for being brave and invited them to talk about their experience of pain. Children exposed to ambiguous communication cried, looked away and physically held on to their parents during the procedure. After the injection, they expressed relief and looked both proud and embarrassed.

#### Disregarding communication

In some cases, both nurses and parents responded to children’s actions rather than their concerns. For example, the following exchange occurred when one child touched the skin after topical anaesthetic was applied:

*Parent: “You shouldn’t have touched it (because it was clean)”*

*Child: “I just wanted to feel … .”*

*Nurse: “It’s okay, we can clean it again” (Case 2)*

This child had shown several signs of fear and tried to become involved during the procedure. The nurse repeatedly turned to the parent and did not respond to the child. When the child shouted out loudly, “*No I don’t want to do it*”, the parent offered to look at the preparation, but the child showed no interest. This child cried for a long time after the injection and reported severe pain. In another case (Table 4), both the nurse and the parents used reassurance to make the child accept the injection. They suggested coping strategies and tempted the child with a reward. This child directly stated severe fear but was not offered sufficient time for reflection and remained afraid.

In these cases, the children gave up their protests, received the injection and expressed more pain than others. They looked sad, and their parents had to comfort them for long time afterwards even though nurses praised the children for their achievement and gave them rewards.

## Discussion

The main finding of this study was that for the most part, children expressed fear indirectly or nonverbally. Anticipatory fear appeared more bothersome than the pain experience itself. We also found that adults’ approach to communication affected children’s opportunity to express their emotions. Children became more involved when nurses acknowledged their fear. Both nurses and parents frequently offered coping strategies unclearly without sufficient time for children to understand.

### Children’s subtle communication of fear and pain

We expected that children would worry about the needles, as the fear of needles is common among children [1, 3]. Prevalence was expected to decrease during adolescence to a range between 20 and 50% [3]. In our study, adolescents’ fears and worries were evident, and these were mainly expressed nonverbally or indirectly. This was in line with previous studies on children in cardiologic and oncologic medical consultations in whom worries were commonly communicated as subtle verbal and nonverbal cues rather than explicit concerns [33, 39]. A *cue* is a verbal or nonverbal hint suggesting an underlying unpleasant emotion lacking clarity, whilst a *concern* may be defined as an explicit expression of a current or recent unpleasant emotion [34]. In the videos, we identified slight smiles, insecure body language, lowered voices or slow movements as typical *cues* of fear. Worries are more likely to be expressed as *cues* than as *concerns*, making them difficult to detect [40]. Therefore, nurses did not always perceive fear until the short interview after the procedure.

The most anxious children reported more intense pain than those who were less anxious, which corresponded with research showing that high levels of fear are associated with increased pain during needle procedures [8]. Pain perception depends on many factors, like how adults behave in the situation and the child’s emotional state and coping skills [24]. Before the procedure, nurses rarely communicated with children about their worries, even though these children were able to describe their emotional state eloquently. Children experienced the stinging pain after the injection as more painful than the needle stick, which emphasised their need for concrete information about this expected pain and a need to have their pain assessed. Systematic assessment of children’s pain and fear, adjusted to their level of maturity, is widely recommended in the literature, and several tools are available for this purpose [12, 41–43]. From a biopsychosocial perspective, acquiring information about patients’ emotional state by identifying cues and concerns is equally as important as gathering information about their physical condition [34]. Our findings suggested that asking children about their worries before a potentially painful procedure gives them an opportunity to verbalise their concerns.

The most anxious children seemed to distance themselves mentally when the injection came closer. They gave up their verbal protests and received the injection, sometimes after repeated persuasion from nurses and parents. These children looked sad, and their body language was stiff or retiring. They avoided looking at the nurse, and they held onto their parents physically. Similar behaviour has been described as surrendering and is one way for the child to regain control during a needle procedure [14]. A study of preschool children who had venepuncture used the term endurance to describe this resistive expression, which occurs after children have given up protesting and escaping [44]. No children in our study tried to escape physically, as they were old enough to understand the reason for the injection. Surrendering behaviour may have been a way for them to prepare for an unpleasant situation, though it may have implied their compliance rather than their acceptance of the procedure [14].

### Adults’ approach to communication

Nurses often paid more attention to details of the needle procedure than to children’s signs of fear. Administering subcutaneous injections to a child is a complex task and requires specific knowledge that may be demanding, especially for nurses who are performing a training session for the first time. Nurses ensured that they selected the correct injection site, the right angle of needle insertion and the right temperature of the medicine per recommendations in the literature [45]. However, their approach to communication may be important for how children express their emotions. Nurses who were able to recognise and understand the role of emotional content in a conversation seemed to form good relationships with both adults and children, which are needed for the development of shared management in medical care [21]. The nurses’ experience was expected to influence their communication, but we observed that some of the less experienced nurses managed the communication very well. This observation may have been related to prior experiences and education that some of these nurses had, rather than their education and experience as nurses.

When nurses had an acknowledging attitude towards children, this provided them with *space* in which to express both positive and negative emotions. Providing space has been explained by healthcare professionals as giving patients the freedom to disclose personal thoughts and feelings while paying attention to their needs and worries [40]. Taking a break provided the child with additional space that seemed to influence them positively. When nurses moved too quickly, even if they recognised children’s fear, the message became ambiguous, even if the content was relevant. When children clearly stated that they were afraid, adults (both nurses and parents) sometimes escalated their number of suggestions. Suggestions became more geared towards persuading children to finish the injection rather than being aimed at reducing children’s distress. Children did not seem to understand or trust these suggestions. Children may have been less distressed if information and a choice of coping strategies had been provided prior to the injection procedure [23, 24].

Acknowledging communication has been characterised by an understanding of children’s perspectives that confirms their experience and by appreciating children’s emotions as well as their actions or achievements [46]. We found that when children were acknowledged, they reached a mutual understanding with the nurse and became more involved in the procedure. These children showed more confidence throughout the procedure. In order to give children essential acknowledgement, nurses must be self-aware during their interactions with children and be sensitive to nonverbal and verbal communication [46]. This is a demanding task, and special competence is required by the nurses. Both children and parents acted more confidently when nurses guided them, showed predictability and took control over what was happening. Other studies have emphasised children’s need for age appropriate information and guidance and have shown that children’s choices should be an integral part of decision making [13, 47].

Both nurses and parents frequently used reassurance (that is, “*It will be okay*” or “*It won’t hurt*”*)* as a natural way of comforting children. This usually did not decrease children’s fear. Adult reassurance has been shown to increase children’s distress during medical procedures [16, 17, 23] and is an example of communication that reduces space for further disclosure compared with a more acknowledging approach [39]. We observed one exception in which parents provided reassurance while the nurse reached a mutual understanding with the child. This child stayed focused and confident. Previous research has emphasised the complexity of reassurance and suggests that adults’ facial expressions, vocal tones and verbal content play an important role in how reassurance is perceived [15].

In the cases characterised by ambiguous or disregarding communication, we observed that children’s confidence increased when afterwards, the nurse or parent(s) reflected on the experience and acknowledged the children’s braveness. Helping children to express their emotions after a painful procedure and shape a more positive memory has a positive influence on later pain experiences [48].

### Children’s willingness to be involved

Children often showed positive engagement when playing with equipment, and some children suggested their preferred coping strategy. However, nurses did not always follow up on these opportunities to form a relationship with the child. Children lack equal opportunities to share their views and participate in decisions regarding their care [49]. Incomplete use of acknowledging communication and coping strategies may explain children’s chances for participation. The children with prior experiences of needle injections appreciated just as much the preparation and training as the other children. Being aware of building this important relationship with every child may prevent the risk of proceeding too fast or skip important steps in the training session.

Appropriate distraction is widely recommended as a way to manage procedural distress [9–11, 47]. In this study, only two children realised that distraction was helpful. Distraction must be experienced as safe and voluntary to be supportive, and children should recognise adults’ actions and believe that they can manage the procedure [13]. The aim of training sessions was to teach children and parents the injection technique. Most children were encouraged to watch the procedure, and they tried to involve themselves even though they were afraid. However, when children are highly anxious, it might be more appropriate to offer distraction and then use a stepwise training schedule for home administration [45]. It seems of utmost importance to assess children’s fear before choosing an appropriate coping strategy. The Distraction in Action Tool (DAT) is a promising screening tool that parents and clinicians have found useful in assessing children’s risk for distress and in teaching distraction techniques that can be used during needle stick procedures in an Emergency Department [50].

Two children were willing to engage in decisional control and managed to self-inject the very first time. They were encouraged and closely guided by nurses, who provided enough time and space. Their parents stayed calm and supportive. Such decisional control and choice between a few options may be appropriate, whilst unclear or open ended suggestions, for example, “*How do you like it?*” may expect too much of children, delay the procedure and leave the child in distress [17].

### Parents need knowledge to support their children during painful procedures

Parents knew that they were supposed to leave the hospital after the training session and administer the next injection at home without any further training. This may have caused them to hesitate or push too hard to finish, so their suggestions and intended emotional guidance were not always perceived by children. Thus, children remained in a state of fear, which is known to undermine the effect of pain-relieving interventions [8]. This challenging situation worried nurses, and as they were unable to offer a follow-up appointment, they advised parents to watch a YouTube video or write down the main messages. Parents are often in a state of shock, fear and disbelief shortly after their child has been diagnosed with a serious disease [51]. Therefore, it may be difficult for them to guide and comfort their child through the procedure. Parents need knowledge and tools provided by competent healthcare providers to support their child and manage their own distress [17, 22].

### Strengths and limitations

To our knowledge, this is the first in-depth study examining training sessions intended to teach the home administration of subcutaneous injections of DMARDs and biologics. Although the sample was small and represented only one single hospital, the present sample contained enough variation in key demographics to identify important patterns related to children’s expression of fear and how adult communication affects children’s emotions. We used video observations to explore real-time actions, producing a valuable foundation for further research and the development of clinical practice.

A limitation is that the video observations only examined scheduled training sessions. We assumed that children repeatedly received informal information about injections during their hospital stay, which could have increased their educational level. In addition, children who met inclusion criteria during the study period might have missed out on an invitation to join the study. Finally, being recruited and filmed engaging in a medical procedure during a busy day at the hospital required extra effort from each nurse.

## Conclusion

Children with rheumatic diseases worry about needle pain and experience the stinging pain that occurs after an injection bothersome. Fear is usually expressed indirectly as cues and nonverbal signs rather than direct statements. When adults acknowledge children’s emotions and offer sufficient coping strategies, children become engaged in the procedure and act confidently. How nurses and parents communicate and interact with children and each other seems essential for children’s coping during the procedure. The initial educational training session may have a great impact on long-term repeated injections in a home setting by providing children with confidence at the onset.

### Implications for clinical practice and further research

Based on these findings, we have suggested that this procedure should be initiated by asking all children (who are able to talk) about their fears and acknowledging their emotions. This simple change may improve children’s experiences of fear and pain during procedures. Small adjustments like these have been significant in shaping children’s future experiences of needle injections [8]. Education on needle injections for home administration requires organisational preconditions like guidelines, informational materials and suitable equipment for training and distraction. To practice technical skills and take care of emotional concerns in one session is a huge challenge, and nurses who have this as part of their job need knowledge and guidance. Most children would probably benefit from having more than one training session with age appropriate preparation, and it may be helpful to assess their fear and use a coping strategy. This may increase their confidence with subcutaneous injections. Further research, such as a larger longitudinal study and the development of a stepwise systematic educational program is warranted.

## Data Availability

The datasets (video recordings and written transcripts) have been stored at Services for sensitive data at UiO and have not been made publicly available. This is due to the high risk of the public identifying the participants, as they have been filmed.

## Abbreviations

DMARDs: Disease-modifying antirheumatic drugs
JIA: Juvenile Idiopathic Arthritis
TA: Thematic analysis
VR-CoDES: Verona Coding Definitions of Emotional Sequences

## References

[1] Taddio A (2012). Survey of the prevalence of immunization non-compliance due to needle fears in children and adults. Vaccine.

[2] McMurtry CM (2016). Exposure-based interventions for the management of individuals with high levels of needle fear across the lifespan: a clinical practice guideline and call for further research. Cogn Behav Ther.

[3] McLenon J, Rogers MAM (2018). The fear of needles: a systematic review and meta-analysis. J Adv Nurs.

[4] Jacobse J (2019). The effect of repeated methotrexate injections on the quality of life of children with rheumatic diseases. Eur J Pediatr.

[5] Tollisen A (2018). Physical functioning, pain, and health-related quality of life in adults with juvenile idiopathic arthritis: a longitudinal 30-year Followup study. Arthritis Care Res (Hoboken).

[6] Guzman J (2015). The outcomes of juvenile idiopathic arthritis in children managed with contemporary treatments: results from the ReACCh-out cohort. Ann Rheum Dis.

[7] Tiller G (2018). Juvenile idiopathic arthritis managed in the new millennium: one year outcomes of an inception cohort of Australian children. Pediatr Rheumatol Online J.

[8] McMurtry CM (2015). Far from "just a poke": common painful needle procedures and the development of needle fear. Clin J Pain.

[9] Uman LS, et al. Psychological interventions for needle-related procedural pain and distress in children and adolescents. Cochrane Database Syst Rev. 2013;10. 10.1002/14651858.CD005179.pub3 24108531

[10] Birnie KA, et al. Psychological interventions for needle-related procedural pain and distress in children and adolescents. Cochrane Database Syst Rev. 2018;4(10). 10.1002/14651858.CD005179.pub4 PMC6517234 30284240

[11] Birnie KA (2014). Systematic review and meta-Analysis of distraction and hypnosis for needle-related pain and distress in children and adolescents. J Pediatr Psychol.

[12] Thrane SE (2016). The assessment and non-pharmacologic treatment of procedural pain from infancy to school age through a developmental Lens: a synthesis of evidence with recommendations. J Pediatr Nurs.

[13] Karlsson K (2016). Experiencing support during needle-related medical procedures: a hermeneutic study with young children (3–7 years). J Pediatr Nurs.

[14] Karlsson K (2016). Consequences of needle-related medical procedures: a hermeneutic study with young children (3–7 years). J Pediatr Nurs.

[15] McMurtry CM (2010). When "don't worry" communicates fear: Children's perceptions of parental reassurance and distraction during a painful medical procedure. Pain.

[16] McMurtry CM, McGrath PJ, Chambers CT (2006). Reassurance can hurt: parental behavior and painful medical procedures. J Pediatr.

[17] Blount Ronald L., Corbin Susan M., Sturges James W., Wolfe Vicky V., Prater James M., Denise James L. (1989). The relationship between adults' behavior and child coping and distress during BMA/LP procedures: A sequential analysis. Behavior Therapy.

[18] Bearden DJ, Feinstein A, Cohen LL (2012). The influence of parent preprocedural anxiety on child procedural pain: mediation by child procedural anxiety. J Pediatr Psychol.

[19] Tates K, Meeuwesen L (2001). Doctor-parent-child communication. A (re) view of the literture. Soc Sci Med.

[20] Jordan A (2018). What adolescents living with long-term conditions say about being involved in decision-making about their healthcare: a systematic review and narrative synthesis of preferences and experiences. Patient Educ Couns.

[21] Dicè F, Doce P, Freda MF. Exploring emotions and the shared decision-making process in pediatrric primary care. Mediterr J Clin Psychol. 2016;4(3).

[22] Taddio A, Rogers JM (2015). Why are children still crying? Going beyond "evidence" in guideline development to improve pain care for children: the HELPinKIDS experience. Pain.

[23] Taddio A (2015). Reducing pain during vaccine injections: clinical practice guideline. Can Med Assoc J.

[24] Blount RL (2006). Pediatric procedural pain. Behav Modif.

[25] Backe-Hansen, E. Barn. The Norwegian National research ethics comitee 2009, last updated 08. February 2016 https://www.etikkom.no/FBIB/Temaer/Forskning-pa-bestemte-grupper/Barn/.

[26] Fossheim, H., J. Hølen, and H. Ingierd, Barn i forskning Etiske dimensjoner, in Forskningsetiske komiteer. 2013, De nasjonale forskningsetiske komiteene:164.

[27] Silverman, D., Interpredting Qualitative Data. 5 ed. 2014, Los Angeles, London, New Delhi, Singapore, Washington DC: Sage Publications Ltd. 456.

[28] Heath, C., J. Hindmarsh, and P. Luff, Video in Qualitaitve Research: Analysing Social Interaction in Everyday Life, ed. D. Silverman. 2010, London: Sage Publications Ltd 170.

[29] Knoblauch H, Schnettler B (2012). Videography: analysing video data as a ‘focused’ ethnographic and hermeneutical exercise. Qual Res.

[30] Braun V, Clarke V (2006). Using thematic analysis in psychology. Qual Res Psychol.

[31] Terry Gareth, Hayfield Nikki, Clarke Victoria, Braun Virginia (2017). Thematic Analysis. The SAGE Handbook of Qualitative Research in Psychology.

[32] Jordan B, Henderson A (1995). Interaction analysis: foundations and practice. J Learn Sci.

[33] Vatne TM (2010). Application of the Verona coding definitions of emotional sequences (VR-CoDES) on a pediatric data set. Patient Educ Couns.

[34] Zimmermann C (2011). Coding patient emotional cues and concerns in medical consultations: the Verona coding definitions of emotional sequences (VR-CoDES). Patient Educ Couns.

[35] Richards, L., Handling Qualitative Data a practical guide. 3 ed. 2015, Los Angeles: Sage 222.

[36] Blikstad-Balas M (2017). Key challenges of using video when investigating social practices in education: contextualization, magnification, and representation. Int J Res Method Educ.

[37] Creswell JW (2014). Research design: Qualitative, quantitative, and mixed methods approaches.

[38] Tong A, Sainsbury P, Craig J (2007). Consolidated criteria for reporting qualitaitve research (COREQ): a 32 item checklist for interviews and focus groups. Int J Qual Health Care.

[39] Vatne TM (2012). Children's expressions of negative emotions and adults' responses during routine cardiac consultations. J Pediatr Psychol.

[40] Piccolo LD (2017). Verona coding definitions of emotional sequences (VR-CoDES): conceptual framework and future directions. Patient Educ Couns.

[41] Birnie KA (2019). Recommendations for selection of self-report pain intensity measures in children and adolescents: a systematic review and quality assessment of measurement properties. Pain.

[42] Ersig AL (2013). Validation of a clinically useful measure of children's state anxiety before medical procedures. J Spec Pediatr Nurs.

[43] McMurtry CM (2011). Children's fear during procedural pain: preliminary investigation of the Children's fear scale. Health Psychol.

[44] Svendsen EJ (2015). Resistive expressions in preschool children during peripheral vein cannulation in hospitals: a qualitative explorative observational study. BMC Pediatr.

[45] Angela C, Carol B (2008). Administering subcutaneous injections to children: what does the evidence say?. J Child Young People's Nursing.

[46] Schibbye A-LL, Løvilie E. Du og barnet. Universitetsforlaget. 2017:155.

[47] Koller D, Goldman RD (2012). Distraction techniques for children undergoing procedures: a critical review of pediatric research. J Pediatr Nurs.

[48] Noel M (2012). The influence of children's pain memories on subsequent pain experience. Pain.

[49] Koller D (2017). Kids need to talk too': inclusive practices for children's healthcare education and participation. J Clin Nurs.

[50] Hanrahan K (2017). The distraction in action tool(c): feasibility and usability in clinical settings. J Pediatr Nurs.

[51] Gomez-Ramirez O (2016). A recurring rollercoaster ride: a qualitative study of the emotional experiences of parents of children with juvenile idiopathic arthritis. Pediatr Rheumatol Online J.

[52] Association, W.W.M., World Medical Association Declaration of Helsinki: ethical principles for medical research involving human subjects. 2013.:4. 19886379

